# Genetic regulation and variation of expression of miRNA and mRNA transcripts in fetal muscle tissue in the context of sex, dam and variable fetal weight

**DOI:** 10.1186/s13293-022-00433-3

**Published:** 2022-05-12

**Authors:** Siriluck Ponsuksili, Eduard Murani, Frieder Hadlich, Alvaro Perdomo-Sabogal, Nares Trakooljul, Michael Oster, Henry Reyer, Klaus Wimmers

**Affiliations:** 1 Research Institute for Farm Animal Biology (FBN), Institute for Genome Biology, Wilhelm-Stahl-Allee 2, 18196 Dummerstorf, Germany; 2 Faculty of Agricultural and Environmental Sciences, University Rostock, 18059 Rostock, Germany

**Keywords:** Fetal weight, Pig, Sex-differences, miRNA, mRNA

## Abstract

**Background:**

Impaired skeletal muscle growth in utero can result in reduced birth weight and pathogenesis of intrauterine growth restriction. Fetal and placental growth is influenced by many factors including genetic, epigenetic and environmental factors. In fact, the sex and genotype of the fetus itself, as well as the mother providing it with a suitable environment, influence the growth of the fetus. Hence, our goal was to decipher and elucidate the molecular pathways of developmental processes mediated by miRNAs and mRNAs in fetal muscle tissue in the context of sex, dam, and fetal weight. Therefore, we analyse the variation of miRNA and mRNA expression in relation to these factors. In addition, the coincidence of genetic regulation of these mRNAs and miRNAs, as revealed by expression quantitative trait loci (eQTL) analyses, with sex-, mother- and weight-associated expression was investigated.

**Methods:**

A three-generation pig F2 population (*n* = 118) based on reciprocal crossing of German Landrace (DL) and Pietrain (Pi) was used. Genotype information and transcriptomic data (mRNA and miRNA) from longissimus dorsi muscle (LDM) of pig fetuses sampled at 63 days post-conception (dpc) were used for eQTL analyses.

**Results:**

The transcript abundances of 13, 853, and 275 probe-sets were influenced by sex, dam and fetal weight at 63 dpc, respectively (FDR < 5%). Most of significant transcripts affected by sex were located on the sex chromosomes including *KDM6A* and *ANOS1* or autosomes including *ANKS1B, LOC100155138* and miR-153. The fetal muscle transcripts associated with fetal weight indicated clearer metabolic directions than maternally influenced fetal muscle transcripts. Moreover, coincidence of genetic regulation (eQTL) and variation in transcript abundance due to sex, dam and fetal weight were identified.

**Conclusions:**

Integrating information on eQTL, sex-, dam- and weight-associated differential expression and QTL for fetal weight allowed us to identify molecular pathways and shed light on the basic biological processes associated with differential muscle development in males and females, with implications for adaptive fetal programming.

**Supplementary Information:**

The online version contains supplementary material available at 10.1186/s13293-022-00433-3.

## Highlights

- Sexual dimorphism of weight and muscle transcript abundance occurs during the prenatal development.
- Most sex-dependently expressed genes are located on the X or Y chromosome.
- Transcript levels associated with fetal weight are enriched in metabolic pathways.
- Coincidence of genetic regulation (eQTL) and variation in transcript abundance due to sex, dam and fetal weight was identified.

**Supplementary Information:**

The online version contains supplementary material available at 10.1186/s13293-022-00433-3.

## Background

Prenatal embryonic and fetal development are important processes closely linked to birth weight and piglet survival, as well as subsequent growth performance and final carcass quality [1]. Birth weight also reflects the nutrient availability to the growing fetus and its ability to utilize these nutrients towards growth and development [2]. In particular, skeletal muscle growth and mass are largely determined during the prenatal period [3–5]. Skeletal muscle appears to be the most affected tissue during fetal adaptations to stress, as glucose oxidation and protein accretion are impaired [6]. Prenatal muscle growth is regulated by complex molecular pathways via various molecules, including microRNAs (miRNA) and their target transcripts. MiRNAs have emerged as key regulators of vital pathways and biological processes, including developmental processes such as myogenesis during prenatal embryonic stages [7–10]. Our previous study identified differentially expressed miRNAs in longissimus dorsi muscle (LDM) between breeds and prenatal stages and revealed several pathways related to muscle development [11]. One of the most studied miRNA, miR-210, which is induced under hypoxia, was also reported in our recent study revealing miR-210 to potentially target genes involved in critical biological processes for muscle growth and fetal development [12–14].

Expression-QTL (eQTL) analysis integrates gene expression levels and genome-wide genotyping information to identify genetic variations associated with changes in gene expression. Data of these different levels, the transcriptome and the phenome, may support each other in evidencing the genetic foundation of traits by being associated with the same genomic region. For instance, our previous study showed that linking eQTL detection and trait-correlated expression data improve the nomination of genes as functional and positional candidate genes for muscle traits. [15]. Variation in complex traits largely reflect polymorphisms affecting regulatory sequences, rather than coding sequences [16]. Knowledge of the position of the genes encoding transcripts and the associated DNA-markers enables the discrimination of cis- and trans-acting eQTL [17]. We have previously shown that the detection of trait-dependent expressed genes in a relevant tissue and eQTL-detection facilitates the identification of genes associated with complex traits such as muscle and meat properties, coping behaviours, immune and metabolic traits; these genes represent strong candidate genes [15, 18–23].

Fetal and placental growth in pigs is influenced by many factors including genetic, epigenetic and environmental factors, even the sex status of adjacent fetuses or the intrauterine position [24]. In fact, the sex and the genotype of the fetus itself which is derived from its parent, are important. The mother further affects fetal growth by not only providing the intrauterine environment, but also by transmission of endocrine drivers, oxygen, gene products, RNAs and proteins to the zygote. In fact, maternal availability of micro- and macronutrients during gestation affects fetal development and implies regulatory adaptive responses at the levels of the proteome, transcriptome, and epigenome [25–27]. It is well-known that genomic imprinting is involved in mechanisms to regulate embryonic and fetal growth. Evidence from mouse suggests that genes that are paternally expressed tend to increase fetal growth, whereas maternally expressed genes restrict fetal growth [28, 29]. Many changes in the prenatal period, including molecular or phenotypic changes, shape birth outcomes in the subsequent generation [30]. For instance, fetal or neonatal programming has been reported when adverse conditions occur during gestation, carrying over adverse effects to late life or even to the next generation [25, 31].

We previously identified differentially abundant miRNAs, and their target mRNAs of LDM samples of fetuses at 63 days post-conception representing extremes in intrauterine growth [13] and the correlation of transcripts levels with fetal weight [14]. Therefore, we aim at further insights into effects, including dam, sex, and variable fetal weight on miRNA and mRNA transcript variation in fetal muscle tissue using a larger set of samples. In addition, we aim to provide genetic evidence for the role of specific transcripts, both mRNAs and miRNA, by discovering eQTL of transcripts and to integrate a genome-wide association study (GWAS) for fetal weight.

## Materials and methods

### Animals and sample collection

Animals and sampling were described previously [13, 14]. The sows were fed twice daily with a complete feed for pregnant sows (Trede und von Pein, GmbH, Itzehoe, Germany) depending on performance and condition, had free access to water and were housed in groups in three-area pens (treading area—slatted floor, lying area—flat covered with litter, feeding stalls—self-catch feeding stalls with partially slatted floor) in the pig facilities of the FBN. F1 sows were mated with F1 boars and slaughtered after 63 days of gestation. Fetal muscle tissue samples were obtained from fetuses derived from F1-sows (*n* = 11) of a three-generation pig F2 population based on reciprocal crossing of German Landrace (DL) and Pietrain (Pi). F2 fetuses (*n* = 118) were extracted from the uteri and their weight was immediately recorded using an electronic scale without any parts of the umbilical cord. Sex was determined by visual inspection. Samples from longissimus dorsi muscle (LDM) were immediately frozen in liquid nitrogen and stored at − 80 °C until RNA extraction. The weight of fetuses at 63 dpc ranged from 78.4 g to 196.6 g. The number of fetuses per dam varied from 5 to 15. All F2 fetuses from this study were from the dam's first litter. For this study, animals did not undergo any experimental treatment, sampling or any other intervention before slaughter. Animal handling as well as the killing was in accordance with the approved guidelines for safeguarding good scientific practice at Research Institute for Farm Animal Biology (FBN) and with the guidelines regarding the protection of animals used for experimental and other scientific purposes in the European Communities Council Directive (86/609/EEC). Animal care and tissue collection procedures were approved by the Animal Care Committee of the FBN.

### RNA isolation and gene expression profiling

Total RNA of LDM of the fetuses was obtained by extraction using Tri-Reagent and subsequent clean-up using the RNeasy Mini kit (Qiagen) with on-column DNase treatment. The RNA integrity and concentration were checked by electrophoresis on 1% agarose gels and in Agilent 2100 Bioanalyzer (Agilent) chips and by spectrometry on a Nano Drop ND-1000 Spectrophotometer (PEQLAB). RNA-expression of LDM from F2 pigs at 63 dpc was assessed using Affymetrix porcine gene expression microarrays (mRNA: GEO platform GPL16569; miRNA: GEO platform: GPL14969). Using 500 ng total RNA isolated from each tissue sample, cDNA of mRNA was synthesized and biotin-labelled using the Affymetrix WT plus Expression kit and Genechip WT terminal labelling and hybridization kit.

Preparation of targets for hybridization from miRNA were generated with the FlashTag™ Biotin RNA Labeling Kit for Affymetrix GeneChip miRNA arrays (Genisphere, Hatfield, PA, USA) starting from 250 ng of miRNA that was poly(A)-tailed using ATP–poly-A-Polymerase. After hybridization, washing, detecting and scanning of the arrays using a Fluidics Station 450 (Affymetrix, Inc., Santa Clara, CA) and a GeneChip^®^ Scanner 3000 (Affymetrix, Inc., Santa Clara, the Expression Console software (Affymetrix) was used for robust multichip average (RMA) normalization. Probe-sets with low signals and present in less than 80% of the samples were removed. For mRNA analyses, 11,288 probe-sets passed the quality filtering and were used; for miRNAs 675 probe-sets were considered for further analysis. The expression data of both miRNA and mRNAs are available in the Gene Expression Omnibus public repository with the GEO accession number GSE169094, GSE162754 and GSE162755.

### SNP genotypes

For genotyping, DNA samples (*n* = 118) were amplified, fragmented, and hybridized to the PorcineSNP60 BeadChip (Illumina Inc., San Diego, CA, USA) containing 62,163 locus-specific 50-mers. Single-base extension of captured oligos with incorporated labels that were detected by an Illumina iScan. Images were subsequently converted to intensity data. Intensity data were normalized and genotypes were assigned using the GenomeStudio software (version 2.0) (Illumina Inc.). Samples with call rates < 99% and SNP-markers with minor-allele frequency < 5% were excluded. The average call rate for all samples was 99.8% ± 0.2. After filtering, 49,036 SNPs were retained for the subsequent GWAS by single-marker analysis approach. The markers of the 60 K chip were mapped to the porcine reference genome, Sscrofa 11.1.

### Expression data analysis in the context of sex, dam and variable fetal weight

In order to reveal variation of gene expression due to fetal weight, sex or dams, normalized expression data served as dependent variables in an analysis of variance. A linear model was used (JMP Genomics 9.0, GLM procedure, SAS 9.4 Software, SAS Inc., Cary, USA) containing the fixed effects (sex and dam) and fetal weight as a covariate. To adjust for multiple comparisons across the Type III tests for all of the effects, the post hoc Tukey–Kramer test was used. To control for multiple testing, adjusted p-values according to Benjamini and Hochberg were estimated and considered significant at 0.1 and 0.05 for miRNAs and mRNAs, respectively [32]. The different significance thresholds used in this study are due to the different number of data used as input (11,288 probe-sets for mRNA and 675 probe-sets for miRNA). Lists of significant probe-sets and associated gene annotations were used for the analysis of canonical pathways in Ingenuity Pathway Analysis (IPA).

### Data pre-processing and eQTL of mRNA and miRNA

After quality control and filtering the expression data were further pre-processed to account for systemic effects. Mixed-model analyses of variance using JMP Genomics (SAS Institute) were used for adjustment. The genetic similarity matrix between individuals was first computed as identity-by-descent of each pair for the k-matrix and used as a random effect. For control of population stratification, top principal components (PC) which explain variation by more than 1% were considered as covariates. In total 18 PCs were included as covariates. Sex was considered as a fixed effect. The residuals were retained for further analysis.

R-package ‘MatrixeQTL’ was used to test the association between each SNP and residuals of transcript abundancies by modelling the effect of genotype as least squares model [33]. ‘MatrixeQTL’ performs a separate test for each gene–SNP pair and corrects for multiple comparisons by calculating FDR [32]. Annotation and localization of SNP sites and probe-sets (Ensembl_Sscrofa_11.1) allowed discrimination of cis- and trans-regulation [34]. We defined an eQTL as ‘cis’ if an associated SNP was located within an area less than 1 Mb from the probe-set/gene. The associations of transcript levels with haematological, biochemical and end production traits were evaluated estimating spearman coefficient of correlation and corrected for multiple comparisons by calculating FDR [32].

### Genomic heritability estimation and GWAS of fetal weight

Genomic heritability represents the proportion of genetic variance explained by SNPs in the phenotypic variance. The estimated genetic influence on the traits was based on the SNP data matrix [35]. The measured SNP-level variation was used to estimate the genetic similarity between individuals, and this estimated genetic similarity was compared to phenotypic similarity to produce a heritability estimate. To estimate the heritability of the fetal weight, sex was included as fixed effect, the genetic similarity matrix between individuals was first computed as identity-by-descent of each pair for the k-matrix (SNPs) used as the random effect.

Fetal weight was analysed for an association with SNPs using a mixed-model analysis of variance in JMP Genomics (SAS Institute, Cary, NC, USA). Mixed-model analysis tests an association between traits and a single SNPs and simultaneously adjusts for population structure and family relatedness [13] which was considered here based on the genetic similarity matrix estimated as a k-matrix. This genome-wide relatedness was used as random effects. For control of population stratification, top principal components (PC) which explain variation of more than 1% were considered as covariates. In total, 18 PCs were included as covariates. Additionally, genotype and sex were used as fixed effects.

## Results

**Fig. 1:**
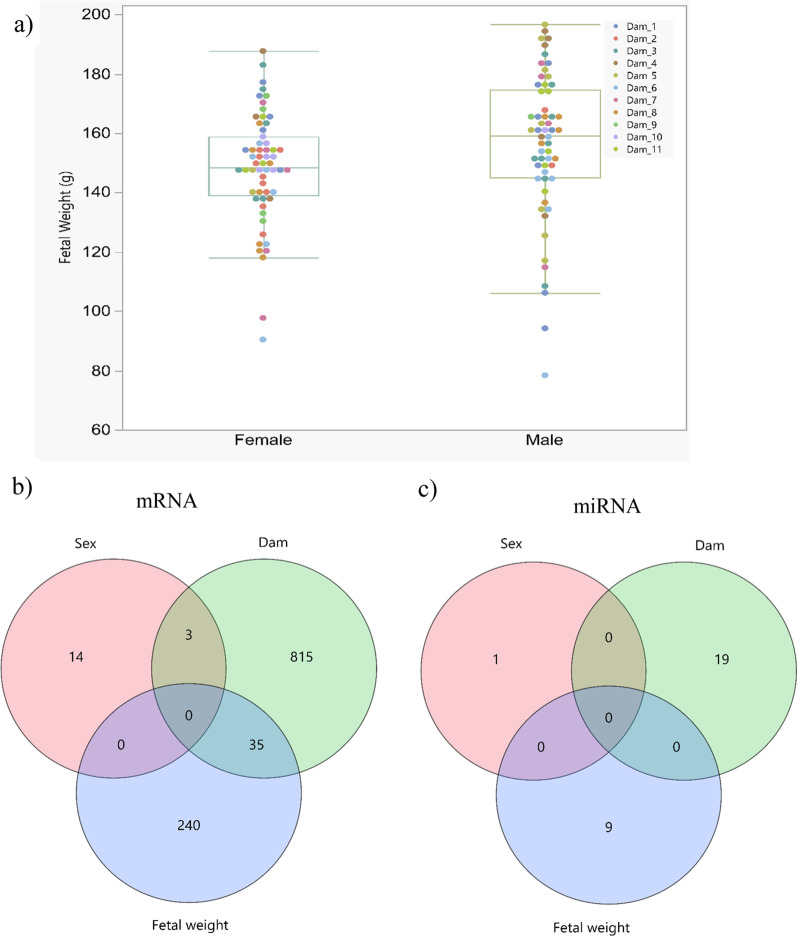
**a** The different of fetal weight at 63 dpc between male (*n* = 58) and female (*n* = 60) (*p* < 0.049) from different dam. Van diagram showing the number of transcripts associated with sex, dam or fetal weight: **b** mRNA transcripts, **c** miRNA transcripts

Fetal weight of 118 samples (58 males and 60 female) from 11 F1 dams mated to one F1 sire was available and averaged 152 ± 22 g (Fig. 1a). The number of fetuses per dam ranged from 5 to 15. When data were analysed with dam as a random effect and sex as a fixed effect, a significant difference in fetal weight at 63 dpc was found between the sexes (*p* = 0.049). The average weight of male and female fetuses was 156 ± 24 and 147 ± 18, respectively.

### Variation of expression of mRNA transcripts due to sex, dam and variable fetal weight

The abundance of 17 probe-sets corresponding with 13 mRNA transcripts was found to be significantly affected by sex. Most of these transcripts were located on the sex chromosomes, except *ANKS1B* and *LOC100155138* that map to chromosome 5 and 14, respectively (Additional file 1). Interesting, *ANOS1* was mapped on chromosome X (SNOWBALL_023609_s_st) and Y (SNOWBALL 020030-s-st and SNOWBALL_020031_s_st). Other transcripts affected by sex were located on the Y chromosome including *LOC100625207* (probable ubiquitin carboxyl-terminal hydrolase FAF-X) and *LOC110255320* (lysine-specific demethylase 6A-like). The transcripts abundance of *LOC110255320* was eight fold higher in male than female. *KDM6A* (lysine demethylase 6A) mapping to chromosome X was higher expressed in female than in male (1.4 fold).

853 and 275 probe-sets were influenced by the dam and fetal weight at 63 dpc, respectively (Additional file 2 and Additional file 3). We further used these genes for IPA pathway enrichment analysis. Top five canonical pathways of dam-associated transcripts belong to Protein Kinase A Signalling, HIPPO signalling, Hepatic Fibrosis/Hepatic Stellate Cell Activation, Epithelial Adherens Junction Signalling and Dilated Cardiomyopathy Signalling Pathway (*p*-value < 10^–5^). The transcripts, which were affected by fetal weight were enriched in the top five canonical pathways of Superpathway of Cholesterol Biosynthesis, TR/RXR Activation, Axonal Guidance Signalling, Cholesterol Biosynthesis I, II and III (p < 10^–4^). One of the KEGG pathways, which reached the threshold of FDR < 5%, is the AMPK Signalling pathway. The transcripts belonging to this pathways included *AKT3* (AKT serine/threonine kinase 3), *FASN* (fatty acid synthase), *GYS1* (glycogen synthase 1), *IGF1R* (insulin-like growth factor 1 receptor), *LEPR* (leptin receptor), *LOC100511937* (phosphatidylinositol 3-kinase regulatory subunit gamma), *PPARGC1A* (PPARG coactivator 1 alpha) and *SCD* (stearoyl-CoA desaturase). In total, we found 14 (9 annotated transcripts), 240 (198 annotated transcripts) and 815 (637 annotated transcripts) probe-sets specific only to sex, fetal weight and dam effect, respectively (Fig. 1b).

### Variation of expression of miRNA transcripts due to sex, dam and variable fetal weight

**Fig. 2:**
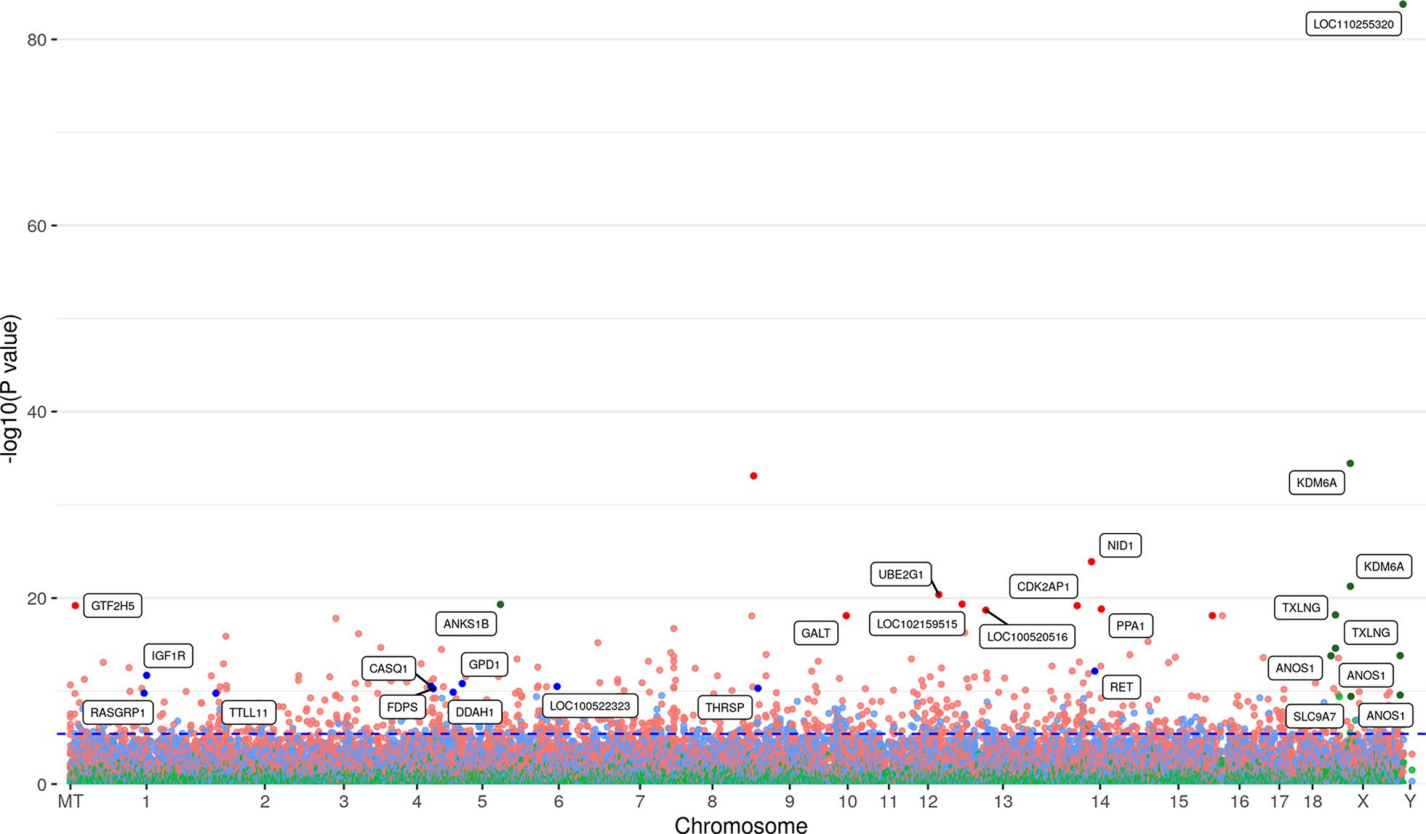
Manhattan plot of the transcript-wide association study of mRNA abundances in muscle samples from 63-dpc-old fetuses (*n* = 118) of an F2 population. The red, blue and green dots indicate the mRNA transcripts associated with dam, weight and sex, respectively. The horizontal dotted line shows the significance threshold of FDR < 0.05 (*y*-axis, -log10 *p*-values). The *x*-axis shows the chromosomal position. Selected significant annotated transcripts are indicated

**Fig. 3:**
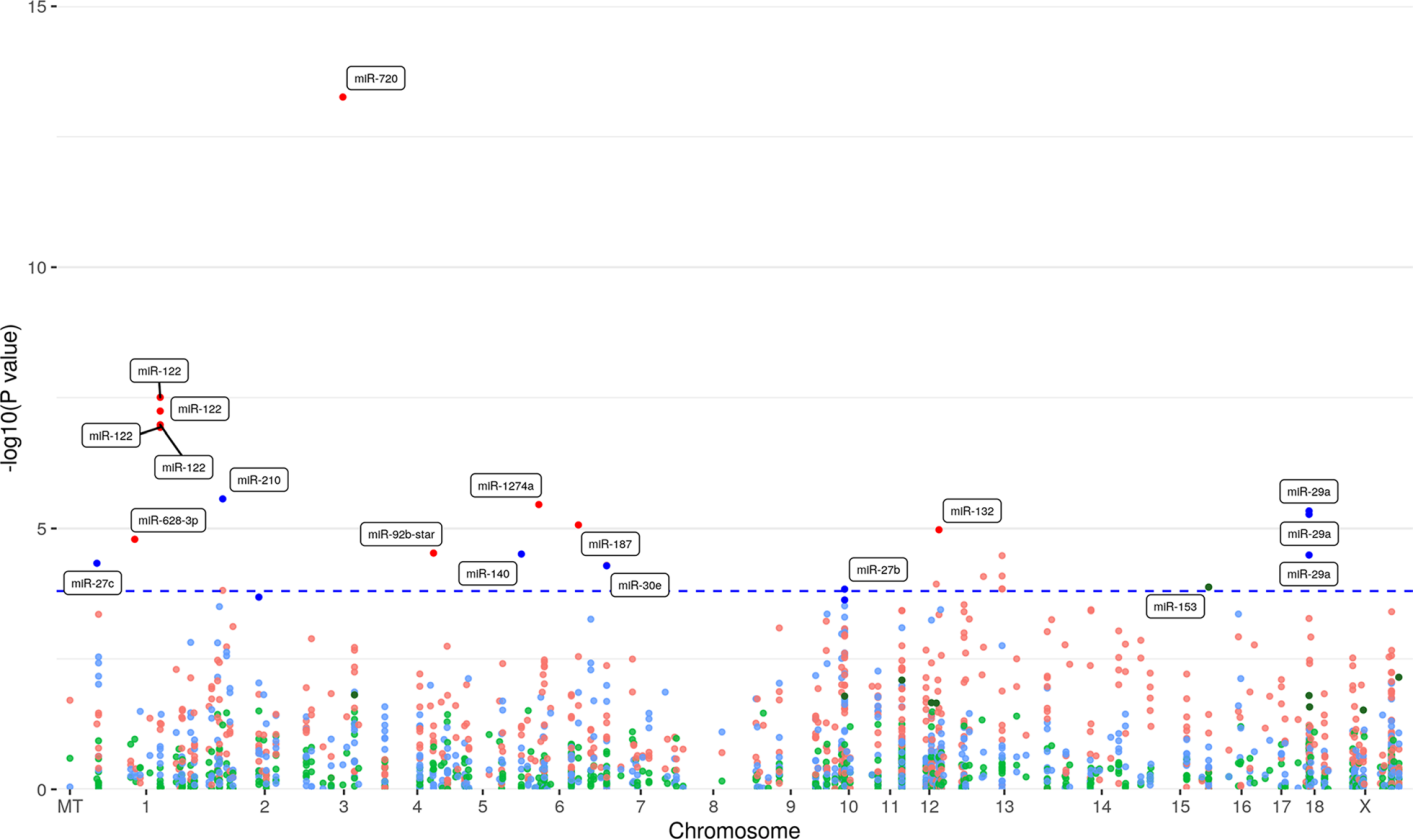
Manhattan plot of the transcript-wide association study of miRNA abundances in muscle samples from 63-dpc-old fetuses (*n* = 118) of an F2 population. The red, blue and green dots indicate the miRNA transcripts associated with dam, weight and sex, respectively. The horizontal dotted line shows the significance threshold of FDR < 0.1 (*y*-axis, -log10 *p*-values). The *x*-axis shows the chromosomal position. Significant annotated transcripts are indicated

Only miR-153 was differentially expressed due to sex. The expression of 19 probes-set corresponding with 13 miRNAs including miR-720, miR-122, miR-1274a, miR-187, miR-132, miR-628-3p, miR-92b*, miR-15b, miR-30c, miR-885-3p, miR-365, miR-16c and miR-210 was influenced by dam and 9 probe-sets corresponding with 6 miRNAs including miR-210, miR-29a, miR-140, miR-27c, miR-27b and miR-30e by fetal weight. No miRNA was found overlapping between effects (Fig. 1c). The manhattan plots showed the genomic position of the mRNA and miRNA transcripts for the different factors (Figs. 2 and 3). Some transcripts are represented by more than one probe-set, for example *KDM6A* (Fig. 2) or miR-122 (Fig. 3).

### mRNA eQTL

**Fig. 4:**
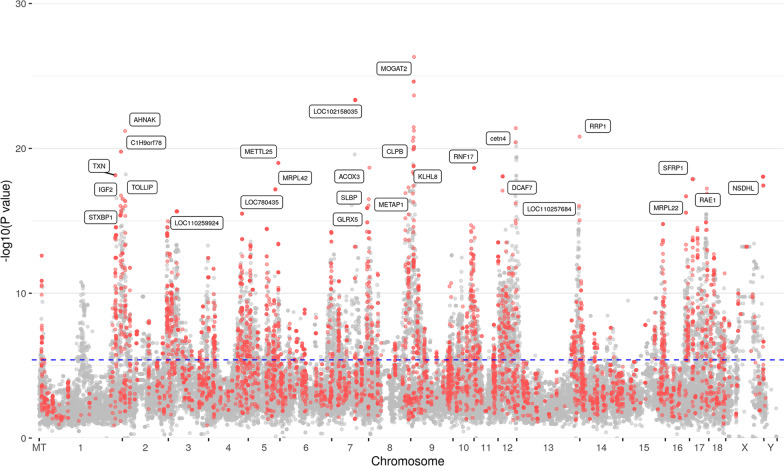
Manhattan plot of the genome-wide association study for mRNA transcript abundances (49,036 SNPs vs. 11,288 mRNA probe-sets). Red dots indicate cis eQTL, gray dots indicate trans-eQTL. The horizontal dotted line shows the significance threshold of FDR < 0.05 (*y*-axis, -log10 *p*-values). The *x*-axis shows the chromosomal position of SNPs. Genes of selected significant eQTL are indicated

A total of 12,622 mRNA-eQTL corresponding to 415 probe-sets (344 annotated mRNAs) were found at *p* < 1.1 × 10^–6^. 9927 mRNA-eQTL which correspond to 342 probe-sets (283 annotated mRNAs) were cis-eQTL. The details of the top 1000 mRNA-eQTL are available in the Additional file 4 and the Manhattan plot (-log10[P] eQTL plot) of the most significant eQTL for each SNP is shown in Fig. 4. Highly significant eQTL were found for many SNPs on SSC 9 with the transcript levels of *MOGAT2* or on SSC11 with transcripts levels of *RNF17.* Most of the significant eQTL were cis-eQTL.

**Fig. 5:**
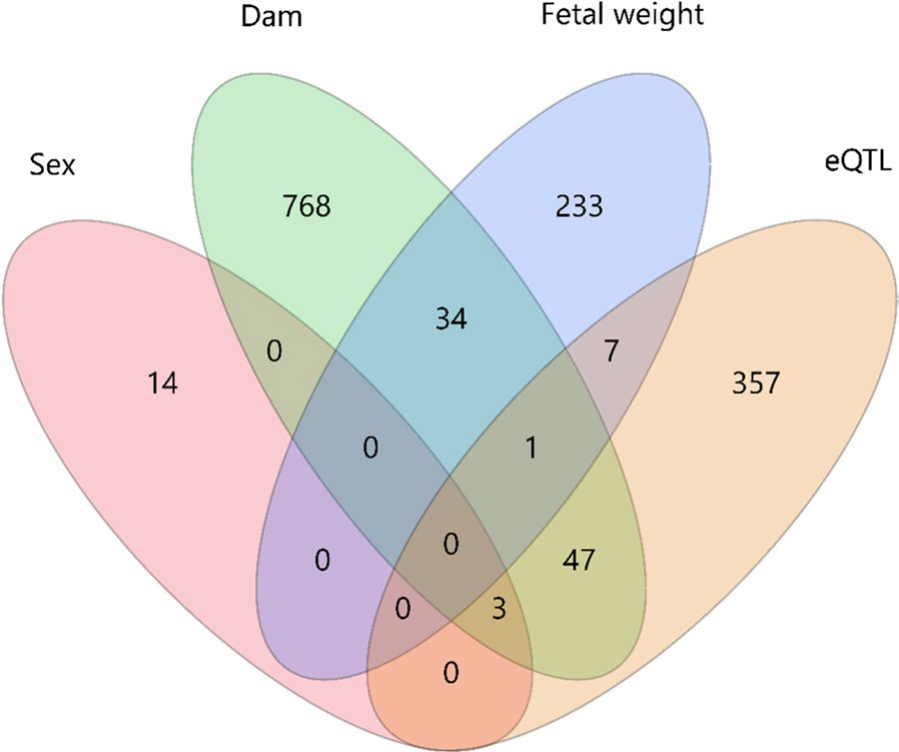
Venn diagram showing the number of transcripts associated with sex, dam, fetal weight and having eQTL

Moreover, 8, 50 and 3 probe-sets showed evidence of genetic regulation (eQTL) and variation of transcript abundance due to weight, dam and sex, respectively (Fig. 5). Seven transcripts were affected by fetal weight and had eQTL including *FXYD2, FIGF, ALDH4A1, LDLR, METTL25, P2RX5* and *AP3M2*. Most of these transcripts had cis-eQTL except *LDLR*. *EHD*1 was the only transcript that was affected by dam and fetal weight and exhibited a cis-eQTL. Three transcripts located on SSC X (*SPIN3, LOC110257707* and *SLC9A7*) were affected by sex and dam and harboured a cis-eQTL. Overlap between dam-associated expression and eQTL effect was found for 47 transcripts.

### miRNA eQTL

A total of 112 miR-eQTL corresponding to 36 miRNAs were found at *p* < 9.8 × 10^–5^ (Additional file 5). Only miR-224 was identified as cis-miR-eQTL which is located on chromosome X at position 123 Mb. H3GA0051468 (rs81473267) located on chromosome X at position 8 Mb was the strongest SNPs associated with many miRNAs including miR-146, miR-122, miR-421, miR-30e-5p, let-7e and miR-217. This SNP is located in the transcript of *ARHGAP6*.

We further investigated whether these genetically regulated miRNAs were also regulated by dam, sex and fetal weight. We found 4 miRNAs (miR-122, miR-132, miR-210, miR-885-5p) among these 36 miRNAs that were also affected by dam and 3 (miR-210, miR-29a, and miR-30e) that were also affected by fetal weight. No significant coincident between sex-associated miRNA and eQTL miRNA were identified.

### Genomic heritability estimation and GWAS of fetal weight

**Fig. 6:**
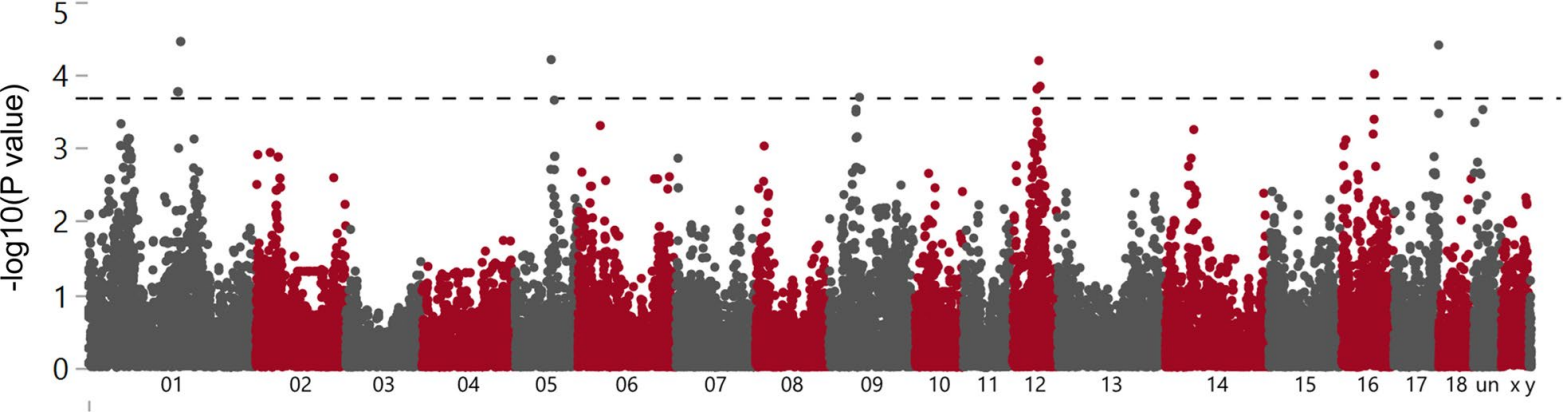
Manhattan plot of the genome-wide association study for fetal weight at 63-dpc-old fetuses (*n* = 118). The horizontal dotted line shows the significance threshold at a *p*-value of 3.7. The *x*-axis shows the chromosomal position; *y*-axis indicates -log10 of *p*-value)

Estimates of genomic heritability of fetal weight were very low (0.06). No SNPs markers associated with fetal weight reached 5% FDR. The top 10 markers associated with fetal weight at p < 1.71 × 10^–4^ are shown in Table 1 and Fig. 6. Most of these markers were located on SSC1 (127.5–132.7 Mb), SSC12 (33.4–38.3 Mb), SSC17 (66.8 Mb) and SSC5 (66.0 Mb).

**Table 1:** Results of genome-wide association studies with single-marker analyses (generalized linear-mixed model) in F2 fetuses (*n* = 118)

SNP_ID	SSC	Position	*p* value	Candidate genes	Major/minor Allele
ALGA0006052	1	132,777,328	3.49E-05	RASGRP1	A/G
ASGA0100924	1	132,615,227	3.49E-05	LOC110261470	A/G
H3GA0002789	1	132,613,568	3.49E-05		G/A
M1GA0022883	17	61,853,177	3.91E-05		G/A
ALGA0032488	5	66,023,540	6.19E-05		A/G
MARC0039239	12	35,751,377	6.39E-05		C/A
M1GA0021029	16	52,624,788	9.77E-05		G/A
ASGA0105561	12	38,311,377	1.43E-04	LOC110255902	G/A
ALGA0120394	12	33,449,301	1.57E-04		G/A
ASGA0004483	1	127,520,993	1.71E-04	FRMD5	G/A

Top 10 markers for fetal weight at 63 dpc

Close to these fetal weight-associated SNPs we found a number of mRNA transcripts that showed variable expression levels due to fetal weight: SSC1 (129–137 Mb) *RTF1, RASGRP1* and *IGF1R*; SSC12 (27–45 Mb) *SPAG9, MSI2, SEPT4, DHX40* and *TAOK1*; SSC5 (62–67 Mb) *RIMKLB* and *TEAD4*. None of the miRNAs that were affected by fetal weight were located in a GWAS region for fetal weight.

Moreover, cis-eQTL for 10 mRNA transcripts (*ACSF2, AKAP1, C12H17orf75, CA4, SCPEP1, SDF2, SGCA, TBX2, TEX14* and *TOM1L1*) on SSC12 (27–45 Mb) co-locate with the QTL for fetal weight on SSC12 (33.4–38.3 Mb). No cis-eQTL were found located close the other QTL for fetal weigth on SSC1 (129–137 Mb). Cis-eQTL of 3 genes (*ERC1, MFAP5, TSPAN11*) were located on SSC5 (62–67 Mb) close to the QTL for fetal weight at SSC5 (66.0 Mb).

## Discussion

The average weight of male fetuses was significantly higher than that of females. The data showed a higher standard deviation in males than in females. These findings could indicate sex-specific development and higher susceptibility of males to disturbed metabolic homoeopathy. Overall, the sexual dimorphism of weight already occurs during the prenatal development.

Knowledge on the effects of sex, dam and variable fetal weight on mRNA and miRNA transcript abundance is still limited in the fetal stage of the pig. Sex-specific differences in miRNA and mRNA expression are reasonable to expect, although few studies have documented these dimorphisms. Studies in mice have found sex differences in skeletal muscle regeneration, such that female cells are more efficient at muscle regeneration [36]. Lysine-specific demethylase 6A (KDM6A) plays an important role in removing repressive histone marks (H3K27), and H3K27 demethylation is required for muscle regeneration and myogenesis [37]. In humans, there are differences in DNA methylation and gene expression between the sexes, including KDM6A in myotubes [38]. In our study, most sex-linked transcripts are located on the X or Y chromosome. The X-linked *KDM6A* is one of them that was found to be more highly expressed in the female muscle than in the male fetus in this study. The higher expression of *KDM6A* in female fetuses compared to male fetuses has also been confirmed in other pig breeds on gestational day 77 [39]. In pigs, myogenesis occurs in two waves of myoblast proliferation (30–60 dpc) and fusion around (54–90 dpc) [40]. In this study, a critical time point was chosen where the formation of primary myotubes and secondary fibres overlaps (63 dpc). In summary, our data show that sexually dimorphic expression of *KDM6A* occurs at the fetal stage and transcript abundance is higher in females. This important epigenetic regulatory transcript may play a significant role in sexual dimorphism during myogenesis in porcine muscle development.

Interestingly, the *ANOS1 (KAL1)* gene, which is an X-linked cause of Kallmann syndrome, was annotated on both the X and Y chromosomes (reference Sscrofa11.1). In our study, two of the three probe-sets of *ANOS1* were located on the Y chromosome and one on the X chromosome. All of them were more highly expressed in male fetal muscles. Sex-specific differences in gene expression have been observed in a number of different tissues, in particular the X- and Y-linked homologues of ANOS1 showed evidence of extreme tissue-specific sex differences [41]. Some transcripts affected by sex including *ANKS1B, LOC100155138* and miR-153 were located on autosome. Sex-specific gene expression on autosomes has also been reported, particularly in the brain at the stage of fetal development [42] or in tissue-specific sex differences [41]. This sex-specific gene expression on autosomes could be due to interactions with sex-specific hormones. Our data shed light on the basic biological processes associated with differential muscle development in males and females, with implications for adaptive fetal programming.

Experimental models of intrauterine growth restriction in farm animals are mostly based on maternofetal stress caused by environmental, nutritional or health conditions [43]. Here we identified more than 800 transcripts of fetal muscle associated with dam including protein kinase A signalling or HIPPO signalling. The HIPPO Signalling pathway is reported to play an early and essential role in mammalian embryogenesis [44]. Disruption of the Hippo Signalling pathway genes in early embryos leads to failure of post-implantation development [44, 45]. In our study, we found that the factor dam also plays an important role in this pathway at the fetal stage. Although more than 800 transcripts of fetal muscle were associated with the dam, functional annotation did not reveal a specific metabolic direction.

Interestingly, the transcripts associated with fetal weight belong to the TR/RXR activation, cholesterol biosynthesis pathway and AMPK pathway. The thyroid hormone receptor (TR), together with the retinoid X receptor (RXR), forms one of the most active complex for gene regulation [46]. We identified the TR/RXR pathway as one of the major canonical signalling pathways associated with fetal weight, including *AKT3, FASN, LDLR, NCOA2, PPARGC1A, SREBF2* and *THRSP*. Thyroid hormone-responsive protein (THRSP) is involved in lipogenic processes and is associated with obesity [47] and differential intramuscular fat in cattle [48]. The Thrsp null mouse demonstrates the role of Thrsp in relation to lipogenesis and obesity [49]. During pregnancy, maternal plasma lipoproteins pass through the placenta and supply lipids to the fetus [50]. In case of lipid deficiency, fetal tissues and the placenta synthesize fatty acids and cholesterol to compensate for a possible lipid deficiency [51]. The changes in *FASN* expressions are in agreement with previously studied associations of placental weight and birth weight with circulating *FASN* and placental *FASN* expression [52]. THRSP, FASN and IGF1R are known transcripts that influence body weight and glucose tolerance in association with increased insulin sensitivity [49, 53, 54]. Cholesterol biosynthesis pathways also play an important role in fetal development and activation of various signalling pathways [55]. In this study, we found transcripts belonging to cholesterol biosynthesis pathways, including *ACAT2, DHCR24, DHCR7, FDFT1* and *FDPS*, to be associated with fetal weight. Many transcripts belonging to these metabolic pathways have been previously identified in the context of intrauterine growth restriction, including *AKT3, FASN, LDLR, NCOA2, PPARGC1A, SREBF2, THRSP, GYS1, IGF1R, LEPR* and *SCD* [13]. Fetal muscle plays an important role in regulating the body's energy balance. Previous studies have shown that gene regulators of fat metabolism in both adipose tissue and skeletal muscle of pigs are associated with birth weight [56]. Together, we showed a link between transcripts involved in TR/RXR activation, cholesterol biosynthesis pathways or AMPK pathway and fetal weight. Compared to transcripts identified as associated with the dam effect, variation in fetal weight is associated with differential expression of genes that relate to metabolic pathways.

Six miRNAs were linked to fetal weight, including miR-210, miR-29a, miR-140, miR-27c, miR-30e and miR-27b. Mir-210 is one of the most studied miRNAs involved in many cellular functions of hypoxia-inducible factor 1-α (HIF1A) under hypoxic conditions [57]. We previously found that miR-210 was upregulated in fetal intrauterine growth restriction and significantly correlated with fetal weight [13, 14]. MiR-210 regulates the expression of oxidative phosphorylation machinery (OXPHOS) and may contribute directly to ATP production [58, 59]. Recently, MiR-210 knockout mice were reported to show its role in regulating placental adaptation to hypoxic stress during pregnancy [60]. One of the factors affecting fetal growth/weight is intrauterine position [24]. Hypoxic conditions can occur with inappropriate intrauterine position/supply and cause alteration in the expression of miR-210 and its targets in the fetus, thus affecting fetal development. Other miRNAs listed here have also been shown to be related to weight and growth. For example, miR-140 was found to be downregulated in the skeletal muscle of fast-growing fish and to be associated with fish body growth [61]. MiR-29a, a member of the miR-29 family of miRNAs, suppresses the expression of insulin-like growth factor 1 (IGF-1) and is thus linked to body weight [62]. The microRNA-29 family dictates the balance between homeostatic and pathological glucose processing in diabetes and obesity [63]. Our previous study identified miR-29 target genes that affect important biological pathways such as bone morphogenesis, placental blood vessel development and ADP/ATP metabolic process, which may lead to impairments in muscle growth and placental development [13]. Target genes of miR-27 are members of the PPAR family, which regulates adipogenesis and obesity [64]. In addition, the miR-23a ~ 27a ~ 24–2 cluster has been reported to downregulate adipogenesis in cattle [65]. Fetal development and pregnancy outcome also depend on the interplay of fetal, maternal and placental mechanisms to protect the fetus from immunological recognition and rejection while providing adequate nutrition. MiR-30e has been reported to regulate NK cell activities at the immune tolerance of the maternal–fetal interface by targeting PRF1 [66]. All these identified miRNA associated with fetal weight show a link between fetal development and a narrow list of candidate transcripts that need further confirmation and verification.

We identified transcripts that are influenced by sex, maternal and fetal weight. We further analysed these mRNAs and miRNAs by looking at the genetic level of regulation. Therefore, we analysed eQTL for both mRNA and miRNA. These molecular traits affect the phenotype at the organism level. Numerous studies have shown that considering expression data of trait-associated transcripts as molecular phenotypes, can support the identification of candidate genes for quantitative organismal traits in GWAS [15, 67]. As in our previous study, a higher number of eQTL were identified for mRNAs than for miRNAs. Moreover, for mRNAs, cis-eQTL dominated trans-eQTL in terms of number and significance [19, 21, 68]. More than 300 annotated mRNA transcripts showed an eQTL effect and more than 80% were cis-eQTL. About 50 transcripts were associated with dam and had an eQTL effect. Three transcripts on the SSC X (*SPIN3, LOC110257707* and *SLC9A7*) were associated with sex, dam and a cis-eQTL. Seven transcripts were associated with fetal weight and had a cis-eQTL effect, including *FXYD2, FIGF, ALDH4A1, LDLR, METTL25, P2RX5* and *AP3M2*. Moreover, miR-224 was the only cis-miR-eQTL found on the X chromosome (123 Mb) in this study. MiR-224 plays an important role in lipid metabolism and targets the 3'-UTR of LPL [69]. Interestingly, three miRNAs, including miR-210, miR-29a and miR-30e, associated with fetal weight were also associated with SNPs defined as trans miR-eQTL. Our previous study showed that highly significant eQTL are associated with high heritability [19]. These results show that the associations between sex, dam and fetal weight on the one hand, and fetal expression on the other, reflect both an underlying effect and a genetic cause and are thus partly heritable.

Most studies focus on birth weight heritability estimates, which range from 0.07 to 0.12 depending on the methods and samples used [70–72]. In this study, the estimated heritability of fetal weight at 63 dpc is also very low (0.06), similar to the heritability of birth weight. In the GWAS of fetal weight, no SNP marker associated with fetal weight reached an FDR of 5%. This is not only due to the small number of samples, but also to the phenotype itself, which is highly influenced by environmental factors, as shown in other studies with larger numbers of samples [70–72]. Previous studies have shown that genetic markers identified by GWAS explain only a limited proportion of heritability [73]. Therefore, it is not easy to identify genomic regions that are most likely to be associated with this trait. Fetal growth is influenced by both genetic and environmental factors, although in this case the environmental influence is more pronounced. The molecular signalling pathways relevant to environmental factors are not yet sufficiently defined. Identifying key genes and pathways that regulate fetal weight will allow better monitoring of intrauterine growth to maximize healthy outcomes. Here, we also used transcript abundance as endogenous traits that are important contributors to the expression of complex traits and depend on genetic polymorphisms, as shown by eQTL analyses. In addition, transcripts associated with birth weights and the genomic region that showed association with fetal weight were integrated. The approach of using eQTL data to support GWAS for the discovery of candidate genes for complex traits has been successfully applied [15, 74]. For example, the QTL region for fetal weight in the SSC1 region 129–137 Mb includes three transcripts (*RTF1, RASGRP1* and *IGF1R*) that also have trait-associated expression and are known to have related functions. Rtf1 is a component of the Paf1 transcription elongation complex, which is required for cardiac muscle development [75]. RASGRP1 is a guanine nucleotide exchange factor involved in the dynamic regulation of the cytoskeleton [76]. Mutation of IGF1R delays intrauterine and later growth in humans [54]. SGCA as a cis-eQTL on SSC12 (27–45 Mb) was another interesting candidate and involved in progressive muscular dystrophies during early human development [77, 78].

No miRNAs whose expression was associated with fetal weight were located in GWAS regions for fetal weight. Further, most of the miRNAs found in this study have trans-eQTL, which means that the SNPs associated with transcript levels are located elsewhere in the genome. Therefore, additional biological information is required to distinguish between causal genes and positional candidate genes of GWAS.

### Perspectives and significance

Our study provides important insights into the effects of sex, mother and variable fetal weight on fetal muscle tissue. Sex-specific expression in muscle was found in the early fetal stage and is mainly due to genes located on the sex chromosome. In the future, more information is needed on sex-specific expression in different organs and fetal stages in relation to hormone production. This information will help to understand the differences in the basic biology of adaptive fetal programming in males and females, the predisposition of individual dams to intrauterine effects on offspring and the variability in fetal weight.

## Conclusions

Analysis of the variation in expression of miRNA and mRNA transcripts in fetal muscle tissue in relation to sex, dam and variable fetal weight revealed that sex-specific expression in fetal muscle is mainly due to genes located on the sex chromosomes, but some autosomal genes also show differential expression due to sex. By shaping the environment of the fetus and providing genes and genetic material, the dam has a major influence on the development of the fetus. Accordingly, the expression of more than 800 transcripts of fetal muscle was associated with the dam, enriched in some canonical metabolic pathway, but overall not suggesting a distinct metabolic orientation of fetal metabolism. Differential expression due to fetal weight was found for genes related to TR/RXR activation, cholesterol biosynthesis pathways or AMPK signalling. Overall, fetal muscle transcripts associated with fetal weight indicate clearer metabolic directions than maternally influenced fetal muscle transcripts. Similar to what is often shown for birth weight, the heritability of fetal weight 63 dpc is also low. Observing the coincidence and colocalization of eQTL, sex-, maternal-, and weight-associated differential expression and QTL for fetal weight allows us to prioritize a number of candidate transcripts affecting fetal development despite the low heritability.

## Supplementary Information

**Additional file 1. **List of transcripts significantly associated with sex at Benjamini & Hochberg (BH-) adjusted p-values < 0.05 for mRNA and < 0.10 for miRNA (shaded grey).

**Additional file 2. **List of transcripts significantly associated with dam at Benjamini & Hochberg (BH-) adjusted p-values < 0.05 for mRNA and < 0.10 for miRNA (shaded grey).

**Additional file 3. **List of transcripts significantly associated with fetal weight at Benjamini & Hochberg (BH-) adjusted p-values < 0.05 for mRNA and < 0.10 for miRNA (shaded grey).

**Additional file 4.** The top 1000 mRNA-eQTL revealed by genome-wide association analyses of miRNA expression values. SNP_IDs and significant associated mRNAs are shown with NegLog10 of p-values and mapping positions. Reported as cis-eQTL when the distance between SNPs and transcripts is < 1 Mb.

**Additional file 5. **112 significant miR-eQTL revealed by genome-wide association analyses of miRNA expression values. miR-eQTL correspond to 36 miRNAs at p < 9.8 × 10^–5^. SNP_IDs and significant associated miRNAs are shown with NegLog10 of p-values and mapping positions. Reported as cis-eQTL when the distance between SNPs and transcripts is < 1 Mb.

## Data Availability

The expression data are available in the Gene Expression Omnibus public repository with the GEO accession number GSE169094, GSE162754 and GSE162755.
